# Altered exosomal protein expression in the serum of NF-κB knockout mice following skeletal muscle ischemia-reperfusion injury

**DOI:** 10.1186/s12929-015-0147-x

**Published:** 2015-06-10

**Authors:** Johnson Chia-Shen Yang, Ming-Wei Lin, Cheng-Shyuan Rau, Seng-Feng Jeng, Tsu-Hsiang Lu, Yi-Chan Wu, Yi-Chun Chen, Siou-Ling Tzeng, Chia-Jung Wu, Ching-Hua Hsieh

**Affiliations:** Department of Plastic and Reconstructive Surgery, Kaohsiung Chang Gung Memorial Hospital and Chang Gung University College of Medicine, No. 123, Ta-Pei Road, Niao-Sung District, Kaohsiung City, 833 Taiwan; Department of Neurosurgery, Kaohsiung Chang Gung Memorial Hospital and Chang Gung University College of Medicine, Kaohsiung City, Taiwan; Department of Plastic Surgery, E-Da Hospital, I-Shou University, Kaohsiung City, Taiwan

**Keywords:** Muscle ischemia-reperfusion (I/R) injury, Exosome, NF-κB, Two-dimensional-gel electrophoresis, Proteomics

## Abstract

**Background:**

The NF-κB signaling pathway plays a role in local and remote tissue damage following ischemia-reperfusion (I/R) injury to skeletal muscles. Evidence suggests that exosomes can act as intercellular communicators by transporting active proteins to remote cells and may play a role in regulating inflammatory processes. This study aimed to profile the exosomal protein expression in the serum of NF-κB knockout mice following skeletal muscle ischemia-reperfusion injury.

**Results:**

To investigate the potential changes in protein expression mediated by NF-κB in secreted exosomes in the serum following I/R injury, the levels of circulating exosomal proteomes in C57BL/6 and NF-κB^−/−^ mice were compared using two dimensional differential in-gel electrophoresis (2-DE), liquid chromatography tandem mass spectrometry (LC-MS/MS), and proteomic analysis. In C57BL/6 mice, the levels of circulating exosomal proteins, including complement component C3 prepropeptide, PK-120 precursor, alpha-amylase one precursor, beta-enolase isoform 1, and adenylosuccinate synthetase isozyme 1, increased following I/R injury. However, in the NF-κB^−/−^ mice, the expression of the following was upregulated in the exosomes: protease, serine 1; glyceraldehyde-3-phosphate dehydrogenase-like isoform 1; glyceraldehyde-3-phosphate dehydrogenase; and pregnancy zone protein. In contrast, the expression of apolipoprotein B, complement component C3 prepropeptide, and immunoglobulin kappa light chain variable region was downregulated in NF-κB^−/−^ mice. Bioinformatic annotation using the Protein Analysis Through Evolutionary Relationships (PANTHER) database revealed that the expression of the exosomal proteins that participate in metabolic processes and in biological regulation was lower in NF-κB^−/−^ mice than in C57BL/6 mice, whereas the expression of proteins that participate in the response to stimuli, in cellular processes, and in the immune system was higher.

**Conclusions:**

The data presented in this study suggest that NF-κB might regulate exosomal protein expression at a remote site via circulation following I/R injury.

## Background

Ischemia-reperfusion (I/R) injury to the skeletal muscle leads to the production of oxygen free radicals, resulting in the occurrence of tissue lipid peroxidation upon re-oxygenation, and release of pro-inflammatory cytokines such as IL-6, IL-1, and TNF-α through the NF-κB signaling pathway. In addition, I/R injury to the skeletal muscle not only affects the muscle but also causes injury to remote organs, which can lead to multiple organ failure and death [1–3]. NF-κB plays an important role in the pathogenesis of I/R injury to the skeletal muscle. Regulation of the initial phase of NF-κB activation provides physiological protection against severe ischemic stress [4, 5]. Selective inhibition of NF-κB has been suggested as a potential therapeutic intervention to treat I/R injury [6, 7]. Moreover, it has been reported that inhibition of NF-κB prevents local and remote organ injury following I/R injury [8].

Exosomes are small, spherical vesicles that are secreted upon fusion of the limiting membrane of multivesicular bodies with the plasma membrane [9]. It has been proposed that exosomes act as intercellular communicators with the carried active cytosolic proteins, mRNAs, and miRNAs [10]. The exosome content can also be transferred into the target cells, either through the direct fusion of exosomes with the cell membrane or through active uptake, which is mediated by endocytosis [11, 12]. The secretion of functional proteins by several cell types, including those of the skeletal muscle [13, 14], has been described as being associated with exosomes [15–17], and is involved in cellular stress responses [18–20] as well as in the modulation of immunological responses [19, 21, 22]. Importantly, the presence of oxidative or hypoxic stress can modulate the expression of biologically active proteins in cell-derived exosomes [9, 20, 22–24], suggesting that stress-related signaling via the exosomes could occur through the transfer of the exosomal protein content.

However, the active circulating exosomal proteins that may potentially participate in the regulation of biological responses following I/R injury to the skeletal muscle remain poorly understood. Moreover, although the inhibition of the NF-κB signaling pathway prevents injury to remote organs following I/R injury [8], it is not known whether there is an NF-κB-mediated change in the expression of circulating exosomal proteins during I/R injury. Therefore, the aim of this study was to profile the change in the expression of exosomal proteins in the serum of C57BL/6 and NF-κB^−/−^ mice following I/R injury to the skeletal muscle, using the complementary techniques of two-dimensional electrophoresis (2-DE) and liquid chromatography tandem mass spectrometry (LC-MS/MS).

## Methods

### Animal handling

Twelve NF-κB^−/−^ (B6.Cg-Nfkb1tm1Bal/J) mice purchased from the Jackson Laboratory (Bar Harbor, ME, USA) and twelve C57BL/6 mice purchased from BioLasco were used in this study (Taipei, Taiwan). The animals were housed in a specific-pathogen-free (SPF) facility that is accredited by the Association for Assessment and Accreditation of Laboratory Animal Care International (AAALAC). All surgical procedures, including analgesia, were performed according to national and institutional guidelines. Animal protocols were approved by the Institutional Animal Care and Usage Committee of the Chang Gung Memorial Hospital (permission number No. 2012091304). Briefly, mice were anesthetized using intraperitoneal injection of an anesthetic cocktail, which consisted of 0.1 mg/g ketamine and 0.01 mg/g xylazine (0.01 mL/g body weight). The anesthetized mice were restrained in the supine position on a heated pad to maintain their body temperature at 37 °C. The model of skeletal muscle I/R injury was performed according to our previous report [25]. The quadriceps muscle was perfused at the femoral artery and was then carefully separated from the femoral bone and from the underlying adductor muscle group. In the ischemic experimental group, ischemia was induced by carefully placing a microvascular clamp across the proximal site of the vascular pedicle for 4 h, after which the clamp was removed. The presence of good vascular flow through the pedicle was verified with direct magnified vision. In the sham-operated group, the muscle was isolated without inducing ischemia with a microvascular clamp. The incision wound was closed with interrupted sutures (4–0 nylon sutures), and the animals were allowed to awaken during the remaining reperfusion time.

### Muscle histochemistry

To evaluate basic muscle morphology after I/R injury, the muscles of sham-operated mice and of experimental mice subjected to ischemia for 4 h and reperfusion for 4 h, 1 day, or 7 days were harvested. The muscles were subsequently covered in Tissue Tek O.C.T. compound (Sakura Finetek Inc., Torrance, CA, USA), frozen in liquid nitrogen–cooled isopentane, and stored at −80 °C until cryosectioning (7 μm). Sections were stained with hematoxylin and eosin.

### Western blotting of nuclear p65

Nuclear protein extracts of the experimental muscles of C57BL/6 and NF-κB^−/−^ mice at 4, 16, and 48 h after I/R injury were used for Western blot analysis using NE-PER extraction reagents according to the manufacturer’s protocol (Pierce Biotechnology, Rockford, IL). Protein extracts (30 μg) were separated on 10 % SDS-polyacrylamide gels, and transferred to nitrocellulose membranes. Membranes were blocked using nonfat milk in Tween-20/Tris-buffered saline (TBST), and incubated with monoclonal rabbit anti-p65 antibody (Cell Signalling) and anti-lamin B1 (Santa Cruz, CA, USA), followed by goat anti-rabbit horseradish peroxidase-conjugated secondary antibodies. Nuclear extract of human umbilical vein endothelial cells (HUVECs) against 10 ug/mL LPS (L3755; Sigma, St Louis, MO) treatment for 24 h was used for positive control of p65 expression according to our previous report [26]. The expression of nuclear p65 protein was assessed against that of lamin B1 using a FluorChem 8900 imaging system (Alpha Innotech, San Leandro, CA) (*n* = 4), and the intensity of each band was quantified using auto-background subtraction during spot density analysis using the AlphaEaseFC software (Alpha Innotech).

### Exosome isolation

For proteomic analysis of the exosome contents, six C57BL/6 mice and six NF-κB^−/−^ mice were used; in both cases, three mice were from the sham-operated group and three from the I/R injury experimental group. Whole blood was drawn from the sham-operated mice and mice subjected to ischemia for 4 h and reperfusion for 1 day, respectively, and collected in RNAprotect Animal Blood Tubes (Qiagen, Valencia, CA, USA) without anticoagulant. The whole blood samples were incubated at room temperature for 15 min and centrifuged at 3000 × *g* for 15 min. Subsequently, white blood cells were carefully removed from the corresponding layers, and the serum (250 μL) was extracted and thawed on ice. The supernatants were transferred to sterile tubes containing 63 μL ExoQuick Precipitation Solution (System Biosciences, Mountain View, CA, USA) and were then mixed. The mixtures were incubated for a minimum of 12 h at 4 °C and were subsequently centrifuged at 1500 × *g* for 30 min at 4 °C. The resuspended exosome pellets were then lysed in a protein lysis buffer.

### Scanning electron microscopy

The exosome isolates were attached to double-sided adhesive tape, fixed to a stage, and then coated with nanogold particles. The exosomes were photographed using a JEOL JSM-5300 scanning electron microscope (Tokyo, Japan) for analysis of size and morphology.

### Proteomic analysis

The exosomes were lysed in lysis buffer containing 2 % sodium dodecyl sulfate (SDS), 1 % Triton-X100, 0.1 M Tris (pH 7.4), and one tablet of Complete EDTA-free protease inhibitors (Roche, Indianapolis, IN, USA). Protein concentrations in the exosome lysates were determined using a BCA protein assay (Pierce, Rockford, IL, USA). The lysis mixture was incubated at room temperature for 60 min and was then centrifuged at 15,000 × *g* for 60 min at 4 °C. Following centrifugation, the resulting supernatant was collected and then quantified with a 2D QUANT Protein Assay Kit (GE Healthcare, Piscataway, NJ, USA). The supernatant containing 300 μg of total cellular protein (20 μL) was mixed with a sample buffer (7 M urea, 2 M thiourea, 4 % CHAPS, 65 mM DTT, 0.2 % ampholytes, and a small amount of bromophenol blue) to obtain a final volume of 450 μL. 2-DE analysis was performed using a 24 cm Immobiline DryStrip (GE Healthcare). Subsequent rehydration followed by isoelectric focusing (set at the highest current, 50 μA/gel, 20 °C) and then SDS-polyacrylamide gel electrophoresis were performed. Following electrophoresis, silver staining, which is compatible with mass spectrometry, was conducted. The gel was scanned using a UMAX Power Look 1100 transmission scanner to obtain images, which were subsequently analyzed with the PDQuest software, version 7.1.0. The protein spots (protein expression with changes greater than twofold, following I/R injury) were excised from the gels and were then subjected to in-gel digestion, after which LC-MS/MS was performed. The resulting data were analyzed using the Mascot database.

### Enzyme-linked immunosorbent assay (ELISA)

To investigate whether the exosomal proteins identified are also present in the serum or the experimental skeletal muscle after I/R injury, expression of a representative exosomal protein, the complement component C3 prepropeptide, which was upregulated in C57BL/6 mice but downregulated in the exosomes of NF-κB^−/−^ mice, was measured. The analysis was performed using exosomes from the sham-operated mice and from mice subjected to 4-h ischemia and reperfusion for 1 d (*n* = 4 for each group). C3 prepropeptide expression was measured by ELISA using a commercially supplied kit (Genway Biotech, San Diego, CA, USA). Briefly, each sample was diluted 1/100,000 in blocking buffer (50 mM Tris, 0.14 M NaCl, 1 % BSA, pH 8.0) and added to the wells of a 96-well plate coated with 100 μl of 2 μg/ml rabbit anti-human C3 prepropeptide. After incubation with 1:10,000 diluted horseradish peroxidase conjugate, 100 μl enzyme substrate 3,3',5,5'-tetramethylbenzidine (TMB) was added for 30 min, then 100 μl of 2 M H_2_SO_4_ was applied to each well to stop the TMB reaction. The absorbance at 450 nm was measured using an ELISA plate reader. Each dilution was measured alongside a set of standards and the results averaged. Results are expressed as micrograms per milliliter (μg/mL) of serum and exosomes or per milligram (μg/mg) of tissue protein.

### Functional analysis of the exosomal proteome

Proteins that had twofold or greater expression and were identified by LC-MS/MS were analyzed using the Protein Analysis Through Evolutionary Relationships (PANTHER; http://www.pantherdb.org) classification system. The PANTHER classification system is a comprehensive method that combines gene function, gene ontology (GO), pathway, and statistical analysis tools to facilitate the analysis of large-scale, genome-wide data from sequencing, proteomics, or gene expression experiments [27]. The PANTHER system allows the prediction of protein classifications based on the proteins’ GO, biological processes, and molecular functions.

## Results

**Fig. 1:**
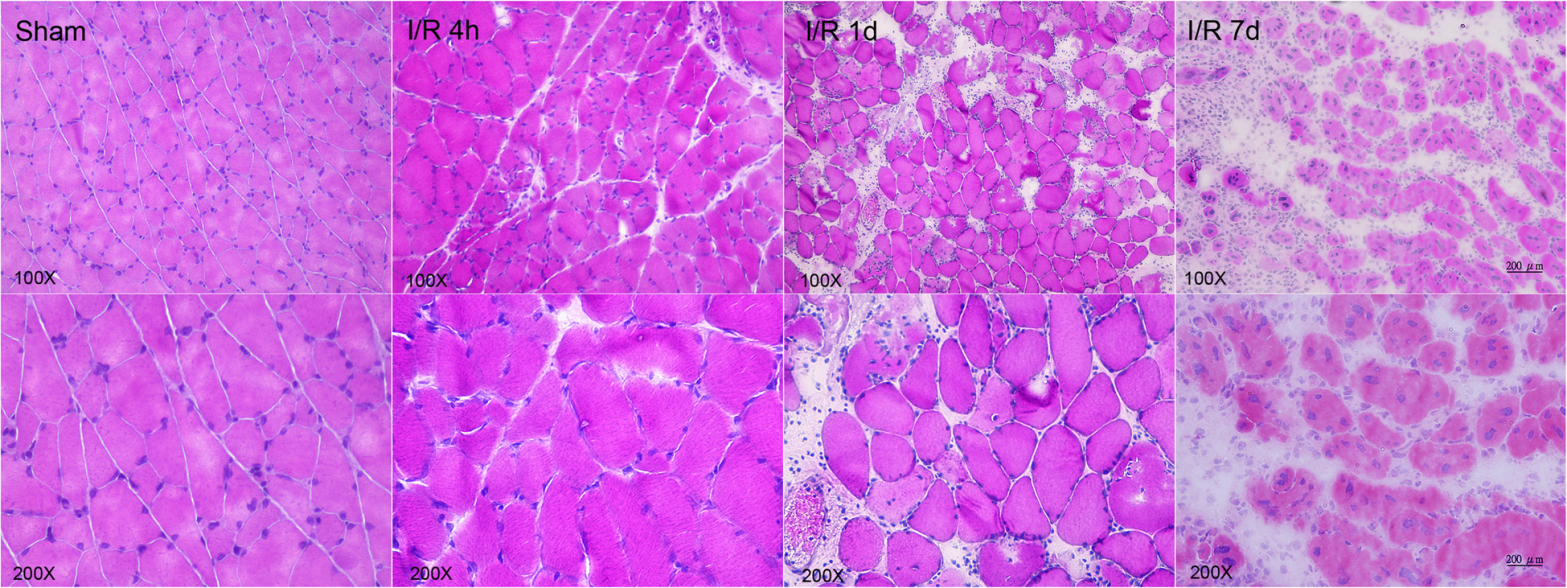
Hematoxylin and eosin staining of the cryosectioned muscles of sham-operated mice and the muscles of experimental mice subjected to ischemia for 4 h and reperfusion for 4 h, 1 day, or 7 days

**Fig. 2:**
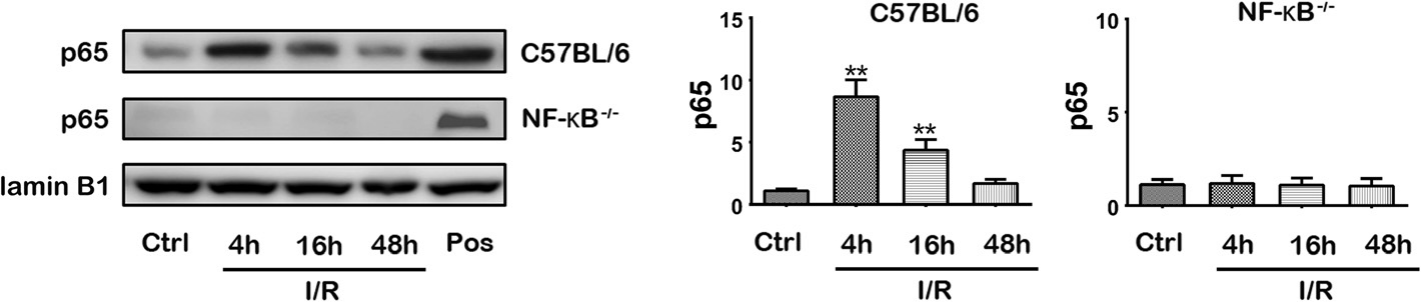
Expression fold of p65 in Western blot analysis of the nuclear protein extracts of the experimental muscles at 4, 16, and 48 h after I/R injury in C57BL/6 and NF-κB^−/−^ mice. (*p < 0.01, compared to sham-operated mice in quadruplicate) Ctrl, Control sham-operated mice; Pos, Positive control of p65 expression

**Fig. 3:**
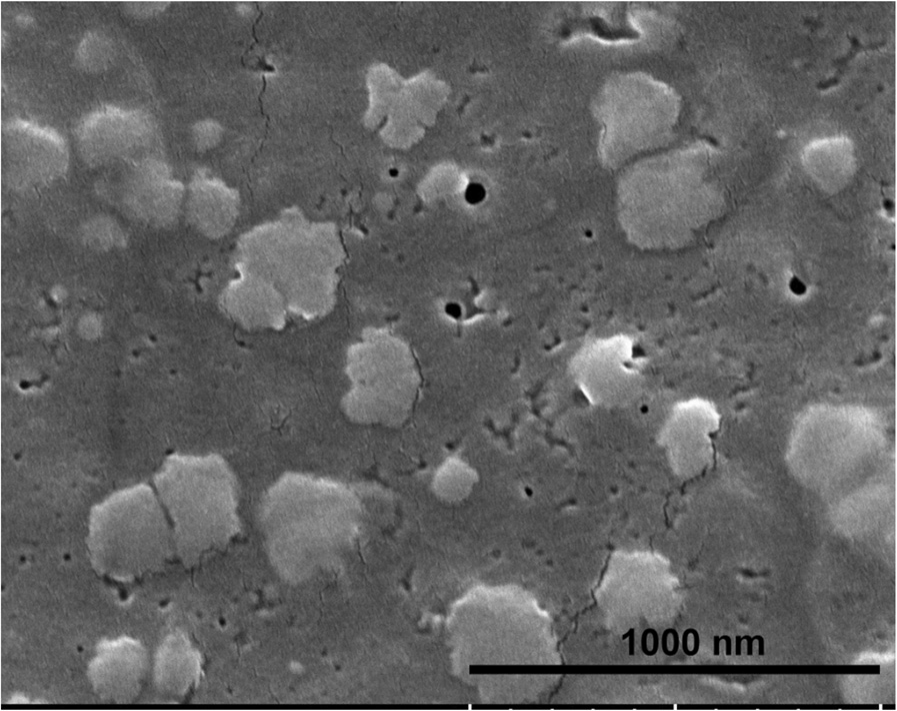
Electron microscopic image of purified serum exosomes derived from a normal C57BL/6 mouse

**Fig. 4:**
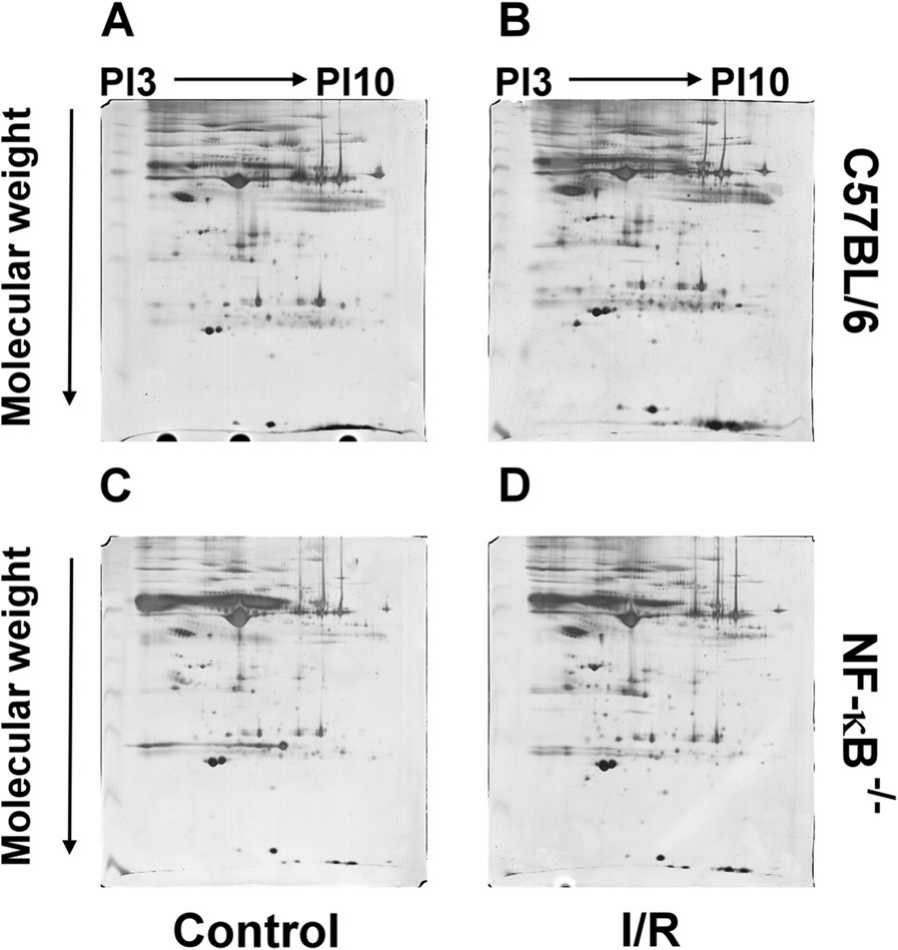
2-DE protein patterns in serum exosomes from C57BL/6 and NF-κB^−/−^ mice after I/R injury. (a) C57BL/6 sham control; (b) C57BL/6 I/R injury; (c) NF-κB-/- sham control; (d) NF-κB-/- I/R injury.

**Fig. 5:**
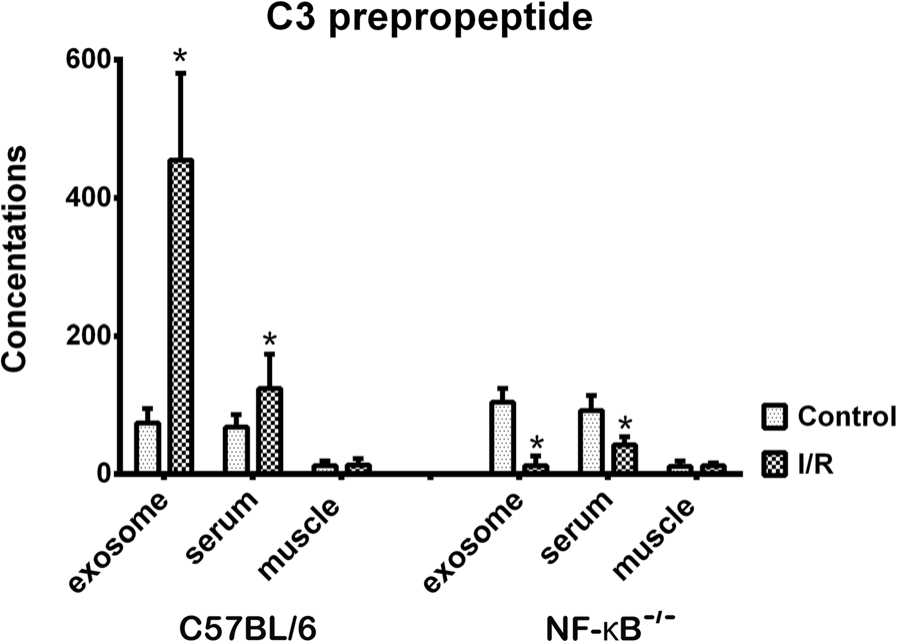
Expression levels of C3 prepropeptide measured by enzyme-linked immunosorbent assay (ELISA) in the exosomes, serum, and experimental skeletal muscle of C57BL/6 and NF-κB^−/−^ mice after I/R injury (*p < 0.01, compared to sham-operated mice in quadruplicate)

**Fig. 6:**
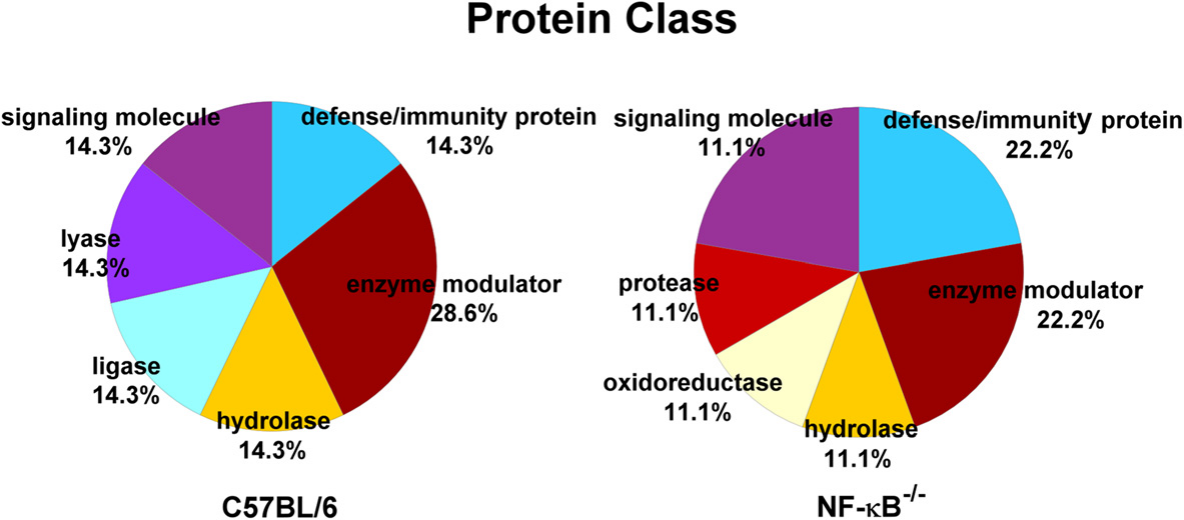
Alteration in serum exosome protein expression after I/R injury. Protein classes were identified and analyzed by mass spectrometry and PANTHER software

**Fig. 7:**
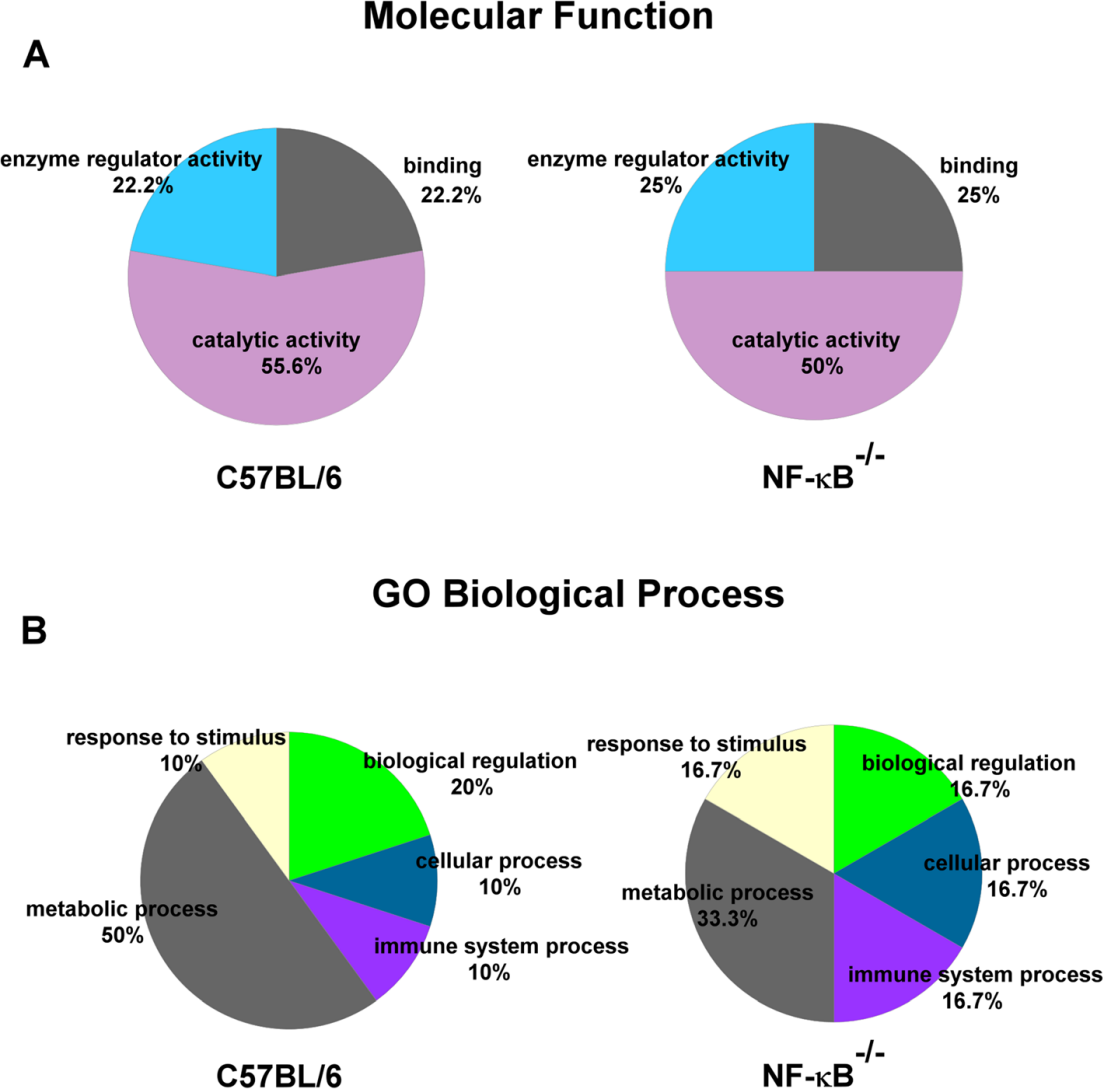
Analysis of exosomal proteins identified by mass spectrometry using PANTHER software. Exosomal proteins isolated from C57BL/6 or NF-κB^−/−^ mice were classified using PANTHER based on (a) their molecular functions and (b) biological processes

Histological analysis involving hematoxylin and eosin staining of the muscles from the experimental group confirmed the presence of I/R injury (Fig. 1). In comparison to the findings in the sham-operated group, increased swelling of muscle fibers with loss of contact integrity among muscle fibers and increased cellular infiltration were found after I/R injury. This morphological change in muscle fibers could still be observed 7 days after the I/R injury. Western blot analysis of the nuclear protein extracts revealed a significant p65 nuclear translocation in the experimental muscle at 4 and 16 h but not 48 h after I/R injury in the C57BL/6 mice. In contrast, no p65 nuclear translocation was found in the NF-κB^−/−^ mice (Fig. 2). The electron microscopy experiments performed using the purified exosome samples obtained from the serum revealed the presence of vesicles that were derived from a C57BL/6 mouse, with an approximate diameter of 50–200 nm (Fig. 3). A proteomics approach involving 2-DE was used to characterize the changes occurring in the serum exosomes following I/R injury and to compare such changes seen in C57BL/6 and NF-κB^−/−^ mice. The resulting protein pattern was observed in a silver-stained 2-DE gel, and a representative image is shown in Fig. 4. The imaging software facilitated the analysis of protein spots on the gel. Protein spots that exhibited a twofold or greater change in expression levels were excised from the silver-stained gels and identified using LC-MS/MS analysis (Table 1). Following I/R injury to the skeletal muscle in C57BL/6 mice, an increase was found in the level of circulating exosomal proteins, including complement component C3 prepropeptide, PK-120 precursor, alpha-amylase 1 precursor, beta-enolase isoform 1, and adenylosuccinate synthetase isozyme 1. However, in the NF-κB^−/−^ mice, the expression of the following were upregulated in the exosomes: protease, serine 1; glyceraldehyde-3-phosphate dehydrogenase (GAPDH)-like isoform 1; GAPDH; and pregnancy zone protein. The expression of apolipoprotein B (ApoB), complement component C3 prepropeptide, and immunoglobulin kappa light chain variable region was downregulated in NF-κB^−/−^ mice. In C57BL/6 mice, the expression levels of C3 prepropeptide in the exosomes and serum after I/R injury were significantly higher than those in the sham group (Fig. 5). However, the fold increase in expression was much higher in the exosomes than in the serum, indicating that the majority of the C3 prepropeptide was located in exosomes. In contrast, the expression level of C3 prepropeptide was significantly lower in both exosomes and serum of the NF-κB^−/−^ mice after I/R injury. There was no significant change in C3 prepropeptide expression in the I/R skeletal muscle in both C57BL/6 and NF-κB^−/−^ mice, indicating that the I/R muscle is not the source of C3 prepropeptide in the exosomes. The data were subsequently subjected to ontology and pathway analyses using the PANTHER software and were then classified based on their respective protein classes, molecular functions, and biological processes. Exosomal proteins that were found to have a greater than twofold change in expression following I/R injury in the NF-κB^−/−^ mice were classified into six GO categories, that is, immunity proteins (22.2 %), enzyme modulators (22.2 %), hydrolases (11.1 %), oxidoreductases (11.1 %), proteases (11.1 %), and signaling molecules (11.1 %). However, in the C57BL/6 group, the functions of the differentially expressed genes consisted of enzyme modulators (28.6 %), hydrolases (14.3 %), ligases (14.3 %), lyases (14.3 %), signaling molecules (14.3 %), and immunity proteins (14.3 %) (Fig. 6). In terms of the molecular function categories, the proteins in both mouse groups that belonged to the functional categories of regulation of enzyme activity, binding, and catalytic activity were highly overrepresented (Fig. 6). In terms of the biological processes, most of the clusters identified were associated with the categories of metabolic processes, responses to stimuli, biological regulation, cellular processes, and immune system processes (Fig. 7). The expression of proteins that participate in metabolic processes and biological regulation was lower in NF-κB^−/−^ mice than in C57BL/6 mice, whereas the expression of proteins that participate in the response to stimuli, cellular processes, and immune system processes was higher.

**Table 1:** Proteins exhibiting a twofold or greater change in expression levels in serum exosomes from C57BL/6 and NF-κB^−/−^ mice in response to I/R injury (*p* < 0.05)

	ID	Name	Score	Expression
C57BL/6	gi|387114	complement component C3 prepropeptide	1963	up
	gi|2739028	PK-120 precursor	2421	up
	gi|160358819	alpha-amylase 1 precursor	2557	up
	gi|6679651	beta-enolase isoform 1	850	up
	gi|6671519	adenylosuccinate synthetase isozyme 1	1577	up
NF-κB^−/−^	gi|16716569	protease, serine, 1	1330	up
	gi|309266468	glyceraldehyde-3-phosphate dehydrogenase-like isoform 1	1346	up
	gi|6679937	glyceraldehyde-3-phosphate dehydrogenase	1075	up
	gi|34785996	pregnancy zone protein	1192	up
	gi|27371137	apolipoprotein B	2617	down
	gi|387114	complement component C3 prepropeptide	2372	down
	gi|158346648	immunoglobulin kappa light chain variable region	3238	down

## Discussion

Our analysis revealed that, during I/R injury, the NF-κB-mediated alterations in the expression of circulating exosomal proteins are related to biological processes that are involved in complement activation, proteolysis, cellular processes, response to stimuli, regulation of catalytic activities, and glycolysis. The putative influences of these proteins on the downstream pathways that are related to I/R include cellular processes, cell communication, and signaling pathways. Based on the classes of exosomal proteins, our data also revealed that the expression of oxidoreductases is upregulated in NF-κB groups, suggesting that the inhibition of NF-κB may modify the expression of the redox modulation enzymes in the exosomes secreted. Oxidoreductases are enzymes that catalyze numerous redox reactions. Their actions include the catalysis of the transformation of free, neutral oxygen gas into oxygen free radicals, superoxide, hydroperoxide, single oxygen molecules, and hydrogen peroxide. They also make up the most important free radical scavenger systems exemplified by catalase, superoxide dismutase, and glutathione peroxidase [28]. Oxidoreductases represent one of the most important free radical scavenger systems and play a cytoprotective role beyond their antioxidant function [29–31]. The possibility of exosomal oxidoreductases in the NF-κB^−/−^ mouse serum contributing to a protective role for the distal organs after I/R muscle injury is interesting and requires further investigation.

The complement system is a host defense system that identifies injured cells, recruits inflammatory cells, and induces cell lysis [32]. The complement component C3 is a pro-inflammatory protein and an important component of the complement cascade. Expression of complement component one is elevated through oxidative stress following ischemic reperfusion [33]. Moreover, many reports have attributed the protective effects of antioxidants to their capacity to suppress the expression of complement component C3 [34–36] and the subsequent blocking of NF-κB activation which inhibits complement component 3 [37]. Importantly, the complement cascade has been implicated in the process of I/R injury. In this study, the expression of the complement component C3 prepropeptide in the serum exosomes of C57BL/6 mice was upregulated in response to I/R muscle injury; however, the expression of this protein was downregulated in NF-κB^−/−^ mice. In addition, although the expression levels of C3 prepropeptide were significantly higher in the exosomes and serum after I/R injury, the fold increase in expression was much higher in the exosomes than in the serum, indicating that most of the C3 prepropeptide was located in the exosomes. Thus, exosomes may participate in inflammatory processes in remote organs during I/R muscle injury; however, such inflammatory effects transmitted by exosomes may decrease during NF-κB inhibition. However, this hypothesis requires further experiments to be validated.

The upregulation of protease, serine one and pregnancy zone protein, an inhibitor of proteinases, was observed in the NF-κB^−/−^ groups, suggesting that the modulation of the function of T lymphocytes and fibrinolysis by these proteins was negatively regulated through the NF-κB pathway following I/R injury [38, 39]. GAPDH, a glycolytic enzyme, has been recently identified as being involved in the initiation of apoptosis [40]. The expression of a phosphorylation-defective GAPDH mutant during I/R injury reduces cell apoptosis [41], suggesting that GAPDH may play a critical role in the progression and spread of ischemic damage [42]. In this study, the expression of exosomal GAPDH was upregulated in the NF-κB^−/−^ groups, suggesting that the expression of exosomal GAPDH is NF-κB-independent and that there may be a negative feedback loop of regulation following I/R injury through NF-κB-independent pathways.

In our study, the expression of ApoB was downregulated in NF-κB^−/−^ mice but not in C57BL/6 mice. ApoB is the primary apolipoprotein of low-density lipoproteins and is responsible for transporting cholesterol to the tissues. The retention and modification of ApoB in the extracellular matrix, followed by proliferation and inflammation, can lead to the chronic progression of atherosclerotic lesions [43]. Alpha-amylase is an enzyme that hydrolyses the bonds of large, α-linked polysaccharides, such as those in starch and glycogen, yielding glucose and maltose. Our results indicated that the expression of alpha-amylase precursor was elevated in C57BL/6 mice. In previous studies, this enzyme has been described as a biomarker of injury to peripheral organs in the serum [44, 45]. Ischemia caused a moderate release of enzymes and an increase in the activity of alpha-amylase [46]. The damage due to ischemia and reperfusion in the pancreas of rats was associated with increased levels of serum alpha-amylase [47]. The elevated plasma levels of alpha-amylase were also investigated in advanced chronic heart failure secondary to ischemic cardiomyopathy [48]. In response to I/R injury, exosomes may serve as carriers of alpha-amylase in the serum and elevation of levels of alpha-amylase in circulation is inhibited by the NF-κB signaling pathway.

Although our data do not provide definitive and exact evidence of the function of the serum exosomes in remote organs following I/R injury, our findings do supplement the current knowledge base regarding their potential function in the regulation of inflammation. The data presented in this study suggest that NF-κB might regulate exosomal protein expression at remote sites via the circulation, following I/R injury.
